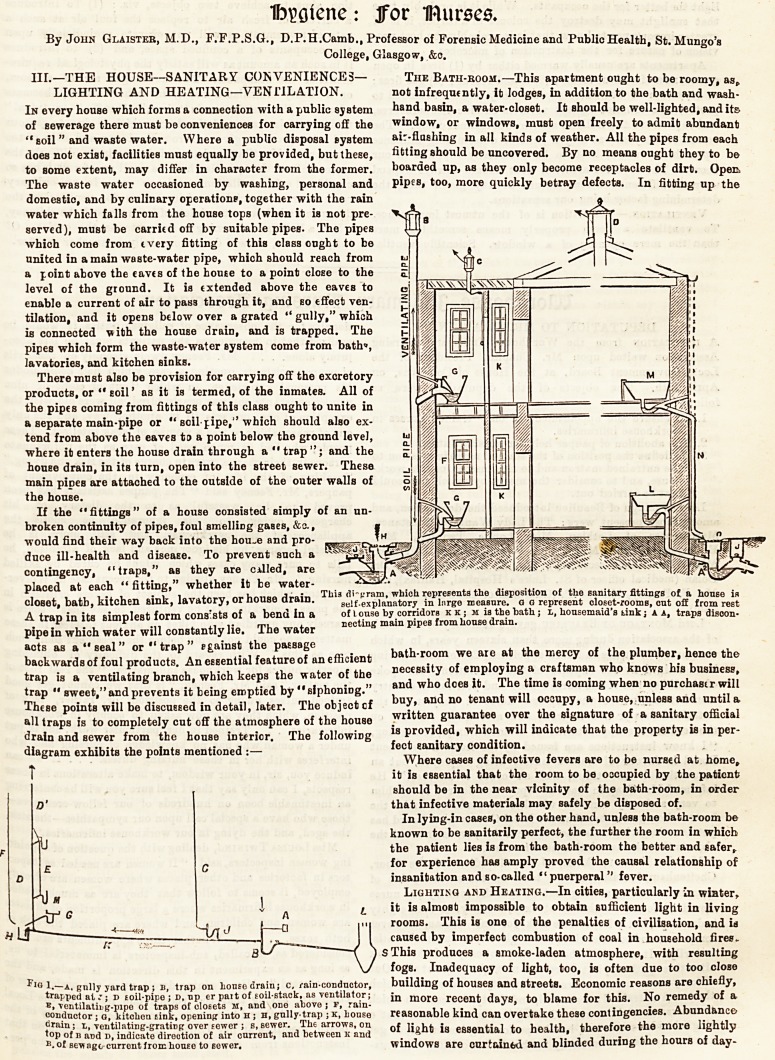# "The Hospital" Nursing Mirror

**Published:** 1896-04-25

**Authors:** 


"The Hospital" Nursing Mirror.

Being the Extra Nursing Supplement of "The Hospital" Newspaper.

[Contributions for this Supplement should be addressed to the Editor, The Hospital, 428, Strand, London, W.C., and should have the word "Nursing" plainly written in left-hand top corner of the envelope.]

### News from the Nursing World.

### FIRST AID AT ALDERSHOT.

The Duke and Duchess of Connaught were much interested in the demonstration of the methods of first aid to the injured, lately held at Aldershot, in connection with the military branches of the St. John Ambulance Association. The Duchess has personally attended the lectures on nursing held at the Officers' Club House by Surgeon-Captain Kilkelly, and both their Royal Highnesses inquired minutely into the various methods of treatment "demonstrated." The transportation of injured men on extemporised litters with the aid of bicycles was a feature of some interest, another being the rescue by the Military Fire Brigade from a burning building. Finally, the Duchess presented certificates to some fifty non-commissioned officers and men, while the Duke did the same to the ladies who had attended the nursing lectures.

### THE EPIDEMIC AT GLOUCESTER.

Affairs do not seem to be much on the mend at Gloucester; the District Nursing Society continues to engage more nurses on its special staff, and has taken a third house for their accommodation. The work of organising all this extra nursing is very heavy, though of voluntary help there seems plenty. Money is the great thing wanted, for subscriptions come in but slowly. The Guildhall is being used as a sort of central store, to which all gifts of food, clothes, old linen, and so forth are sent, and from which the nurses are empowered by the sanitary authority to ask for the necessaries they need in their battle with the epidemic.

### MIDWIVES IN PHILADELPHIA.

The text of a recent "Act of Assembly" for the prevention of blindness in new-born children has been issued by the Philadelphia Board of Health, in the form of an instruction to "midwives, nurses, and others having the care of new-born infants." By this Act, a report in writing has to be made to either the health officer or a legally-qualified practitioner, of any inflammation, swelling, or redness of one or both eyes in an infant at any time within two weeks after birth. On the receipt of this report, the medical officer is to notify the parents of the infant of the danger to the eyes, and enclose directions for proper treatment. Failure to comply with the provisions of the Act is punishable by a fine, "not exceeding 200 dollars, or imprisonment not exceeding thirty days, or both."

### NURSING IN SCOTLAND.

The Ayr Sick Poor Nursing Association has just celebrated its twenty-first birthday, and at a public meeting held on that occasion eloquent testimony was unanimously borne to the value of the work it has been steadily accomplishing during these years. It was agreed on all sides that a second nurse had become a necessity, and increased subscriptions would be needed to allow of the taking of a larger house, and to defray the cost of furnishing and other expenses. Dr. M'Cree paid a well-deserved tribute to Nurse M'Callum's "marvellous activity, boundless energy, and apparently unlimited capacity for overtaking the ground in her visits," of which she paid "no fewer than 4,219 last year." Some good speeches were made, notably by Dr. Naismith and Dr. Rowand, who both dwelt on the very great power and responsibility which rests on district nurses as the diffusers of practical education amongst the poor. The popularity of the association in Ayr is evidently such that no difficulty will be experienced in obtaining the funds needed to supply a second trained nurse with all speed.

### NEW NURSES' HOME FOR WORCESTER INFIRMARY.

The one hundred and fiftieth anniversary of the Worcester Infirmary was celebrated last week by a public dinner, at which a special appeal was made for funds to build a proper Home for the nursing staff. £2,500 is the full amount asked for, and of this £900 was subscribed on the spot. The Lord Lieutenant, Lord Coventry, presided, and Lady Coventry, the Mayor, Lord Beauchamp, Lord and Lady Hindlip, the Dean of Worcester, and Mrs. Forest were also present. The various speeches made showed a universal feeling that the proposed building is only a due recognition of the nurses' services and an urgently needed step. Mr. Bates, a member of the medical staff, remarked that "they were only doing what well became them in endeavouring to provide a good home for their nurses. The wear and tear on the nurse was much greater than it used to be, and so she needed, just as doctors did, a home and rest, a retreat where she could refresh and re-equip herself for the next day's work."

### YET AGAIN!

The usual thing has happened, this time at the Liverpool Workhouse. A patient, a woman aged 35, had been suffering from "pains in her head, and had been strange in her actions." On the night of April 11th she left the ward in her nightdress, and in a minute or two later was "found lying in the basement, having fallen from one of the landings." The injuries received were so severe that she died the following day. At the inquest it was stated that "there was no nurse on duty in the ward from half-past nine to half-past ten on Saturday night," the explanation being that there was only one nurse on each floor, and that on that particular floor there were no less than six wards! There was "a woman" in charge of each ward who summoned the nurse when required. It had not been thought that this particular patient needed special attention. The jury returned a verdict of "Suicide whilst temporarily insane," and gave their opinion that "sufficient care had not been exercised." The master of the workhouse said the vestry were "about to consider the question of increasing the staff of nurses," to which the coroner replied "he thought it high time something was done in that way." Comment is needless.

### SANITARY PRECAUTIONS IN SCOTLAND.

The dangers of circulating libraries in connection with the spread of contagious disease are more real than many people imagine, in spite of the penalties levied upon transgressors, for in England it is comparatively easy for these to escape detection. "Over the border," however, the efforts to stop this particular form of dissemination of germs are more vigorous, and in Edinburgh such strict supervision has been adopted that there is no chance for the convalescing sufferer from scarlet fever or measles to obtain mental distraction during weary weeks of recovery through the medium of the libraries. Every day the library authorities are notified of the names and addresses of all people suffering from illnesses which may be conveyed by books, and the records are examined to see if any such persons, or others living in the same house, are in possession of books. If this is the case, word is sent to the sanitary authority, by whom they are confiscated, and either disinfected thoroughly, or, if necessary, destroyed. Such care is needful enough, while the public conscience is so very elastic on this score, but the organisation entailed must be enormous in a large city.

### MIDWIVES REGISTRATION BILL.

Dr. Robert Boxall and Mr. Rowland Humphreys, hon. secretaries of the Midwives Registration Association, have addressed a letter to the *Lancet* and the *British Medical Journal*, reminding "those members of the profession who are in favour of the education, registration, and supervision of midwives, that a Bill for securing these objects has been set down for second reading in the House of Commons on May 6th." They point out the need for bringing the question under the notice of members of Parliament, and the fact that a number of medical men are in favour of the Bill, urging the advocates of legislation to give active support to this present measure, "which is identical with the amended Bill of Lord Balfour of Burleigh, introduced and read a second time in the House of Lords last year."—The Council of the British Medical Association resolved at their session, April 15th, to "ask the Lord President of the Council to receive a deputation of the Association to urge the Government to oppose the Midwives Registration Bill on the following grounds: (1) That it abrogates the existing Medical Acts as regards the practice of midwifery. (2) That it is fraught with danger to the public, inasmuch as it contemplates the creation of an inferior order of pracittioners to attend upon the poor. (3) That it is unjust to the medical profession, inasmuch as it proposes to register an inferior order of practitioners, instead of a body of well-qualified midwifery nurses. It was also decided that a petition should be prepared against the Bill for presentation to the House of Commons.

### A WISE PRECAUTION.

It has been recommended to the City Council by the Health Board of St. Louis, Missouri, U.S.A., that an ordinance shall be adopted in that town making it a punishable offence for any person "to expectorate on the floors of street cars, or public conveyances, or in places of public resort such as theatres, hotel corridors, or churches," and that those in charge of the conveyances and public places shall be required to supply a sufficient number of spittoons, and to clean these vessels daily, using for the purpose some disinfecting fluid to be approved by the Board of Health. It is also proposed that arrangements shall be made for due inspection to ensure the proper carrying out of the regulations. Travellers in London omnibuses and on the underground railway will wish that some similar enactment might become law in our own country, and so get rid of a nuisance which is dangerous as well as disgusting, here as in America.

### NEWCASTLE NURSES' HOME.

The Nurses' Home and Training School, Granville-road, Newcastle-on-Tyne, is a very successful institution, and numbers now no less than 70 nurses and 10 probationers on its staff. During the past year 618 cases were nursed, the earnings amounting to £3,590, and a sum of £305 having been divided amongst the staff as bonus money. The committee are desirous of accumulating money to enable them to help their nurses to provide against illness or old age, for which purpose they would gladly accept any donations or presents. We are glad to see that the advantages offered to nurses by the Royal National Pension Fund are fully set forward in the annual report, and no doubt when the committee are able to carry out their intention of forming this proposed fund for the relief of their nurses, when from illness or other causes they are unable to work, it will take the shape of affiliation with that organisation.

### SHORT ITEMS.

A fancy fair in aid of the re-endowment fund of Guy's Hospital is to be held in May, under the patronage of the Princess of Wales, in the grounds of Shortlands House, Shortlands, kindly lent by Mr. L. P. Ford.—Lady Cadogan and Lady Sophie Cadogan recently paid a long visit to Dr. Steevens' Hospital, Dublin. They were received by the medical and nursing staff, and escorted round the wards, afterwards going to the board-room, where there were for inspection some interesting curiosities connected with the hospital, amongst others the original roll of subscribers' names, including Dean Swift's autograph.—By the will of the late Mr. John Death, of Poplar House, Cambridge, amongst other charitable bequests (notably £2,000 to Addenbrook's Hospital), the sum of £500 is left to the Town Fund of the Home and Training School for Nurses, Fitzwilliam Street, Cambridge.—A new district nursing association has been started at Buntingford which is to be affiliated to the Queen Victoria's Jubilee Institute, and will, it is hoped, ultimately extend its help to several surrounding parishes.—Miss Priscilla Wild has been appointed district nurse at Kirkley, near Lowestoft.—At the last monthly meeting of the Penrith Women's Nursing Association an interesting lecture was given by Miss Carson on "Children's Ailments, and how to know when the symptoms were serious, with simple directions as to treatment."—Mrs. Makropoulo, the English nurse who has been in the service of the Royal Family of Greece since the birth of the Duke of Sparta in 1868, has died quite suddenly. At the conclusion of the funeral service, held by the English chaplain, the King himself, with the Duke of Sparta and Princes George and Nicholas of Greece, carried the coffin to the hearse, afterwards following it on foot to the cemetery.

### Hygiene: For Nurses.

By John Glaister, M.D., F.F.P.S.G., D.P.H.Camb., Professor of Forensic Medicine and Public Health, St. Mungo's College, Glasgow, &c.

### III.—THE HOUSE—SANITARY CONVENIENCES—LIGHTING AND HEATING—VENTILATION.

In every house which forms a connection with a public system of sewerage there must be conveniences for carrying off the "soil" and waste water. Where a public disposal system does not exist, facilities must equally be provided, but these, to some extent, may differ in character from the former. The waste water occasioned by washing, personal and domestic, and by culinary operations, together with the rain water which falls from the house tops (when it is not preserved), must be carried off by suitable pipes. The pipes which come from every fitting of this class ought to be united in a main waste-water pipe, which should reach from a point above the eaves of the house to a point close to the level of the ground. It is extended above the eaves to enable a current of air to pass through it, and so effect ventilation, and it opens below over a grated "gully," which is connected with the house drain, and is trapped. The pipes which form the waste-water system come from baths, lavatories, and kitchen sinks.

There must also be provision for carrying off the excretory products, or "soil' as it is termed, of the inmates. All of the pipes coming from fittings of this class ought to unite in a separate main-pipe or "soil-pipe," which should also extend from above the eaves to a point below the ground level, where it enters the house drain through a "trap"; and the house drain, in its turn, open into the street sewer. These main pipes are attached to the outside of the outer walls of the house.

If the "fittings" of a house consisted simply of an unbroken continuity of pipes, foul smelling gases, &c., would find their way back into the house and produce ill-health and disease. To prevent such a contingency, "traps," as they are called, are placed at each "fitting," whether it be water-closet, bath, kitchen sink, lavatory, or house drain. A trap in its simplest form consists of a bend in a pipe in which water will constantly lie. The water acts as a "seal" or "trap" against the passage backwards of foul products. An essential feature of an efficient trap is a ventilating branch, which keeps the water of the trap "sweet," and prevents it being emptied by "siphoning." These points will be discussed in detail, later. The object of all traps is to completely cut off the atmosphere of the house drain and sewer from the house interior. The following diagram exhibits the points mentioned:—

Fig 1.—A, gully yard trap; B, trap on house drain; C, rain-conductor, trapped at J; D soil-pipe; D, upper part of soil-stack, as ventilator; E, ventilating-pipe of traps of closets M, and one above; F, rain-conductor; G, kitchen sink, opening into H; H, gully-trap; K, house drain; L, ventilating-grating over sewer; S, sewer. The arrows, on top of B and D, indicate direction of air current, and between K and B, of sewage-current from house to sewer.

The Bath-room.—This apartment ought to be roomy, as, not infrequently, it lodges, in addition to the bath and wash-hand basin, a water-closet. It should be well-lighted, and its window, or windows, must open freely to admit abundant air-flushing in all kinds of weather. All the pipes from each fitting should be uncovered. By no means ought they to be boarded up, as they only become receptacles of dirt. Open pipes, too, more quickly betray defects. In fitting up the

This diagram, which represents the disposition of the sanitary fittings of a house is self-explanatory in large measure. G G represent closet-rooms, cut off from rest of house by corridors K K; M is the bath; L, housemaid's sink; A A, traps disconnecting main pipes from house drain.

bath-room we are at the mercy of the plumber, hence the necessity of employing a craftsman who knows his business, and who does it. The time is coming when no purchaser will buy, and no tenant will occupy, a house, unless and until a written guarantee over the signature of a sanitary official is provided, which will indicate that the property is in perfect sanitary condition.

Where cases of infective fevers are to be nursed at home, it is essential that the room to be occupied by the patient should be in the near vicinity of the bath-room, in order that infective materials may safely be disposed of.

In lying-in cases, on the other hand, unless the bath-room be known to be sanitarily perfect, the further the room in which the patient lies is from the bath-room the better and safer, for experience has amply proved the causal relationship of insanitation and so-called "puerperal" fever.

Lighting and Heating.—In cities, particularly in winter, it is almost impossible to obtain sufficient light in living rooms. This is one of the penalties of civilisation, and is caused by imperfect combustion of coal in household fires. This produces a smoke-laden atmosphere, with resulting fogs. Inadequacy of light, too, is often due to too close building of houses and streets. Economic reasons are chiefly, in more recent days, to blame for this. No remedy of a reasonable kind can overtake these contingencies. Abundance of light is essential to health, therefore the more lightly windows are curtained and blinded during the hours of daylight the better for the occupants. While it is probably true that sunlight may destroy the colours of a carpet, it is of greater importance to know that it is probably the best provision of nature for the destruction of microbes.

Apartments are usually warmed either by (1) fires in open grates, now of various forms; (2) closed stoves; (3) gas fires; or (4) steam or hot-water pipes. They are all intended to protect the inmates against variable outside temperatures, and to preserve the walls of apartments against damp. The season of the year has only a general determining influence upon the amount of heat to be artificially supplied in our rooms. The need for artificial heat should always depend upon the difference of temperature at any given time, the determining factor being our sensations.

Ventilation.—Ventilation is of the utmost importance. To ventilate a room properly means something more than the mere opening of a window. Scientific ventilation aims to achieve two objects, viz.: (1) To introduce a current of fresh air to replace the foul air at such a rate of movement as will not operate prejudicially upon the occupants of a confined space, and (2) to introduce it in such an amount as will satisfy the physiological requirements of the occupants. The unchanged air of a room, or a badly ventilated room, in other words, becomes odorous. We use the words "close," "stuffy," or "foul" to denote this characteristic odour, which is due to gaseous and other products given off from the lungs and skin of the occupants. Ventilation becomes increasingly difficult the more confined the space is; hence a small apartment is not so capable of safe ventilation as a large one. In an ordinary room where no special provision exists for ventilation the doors and windows act as inlets of fresh air, and the chimney, where a live fire is burning in the grate, as the outlet. Of this, however, we will deal more fully in another chapter.

### Workhouse Infirmary Nursing Association.

### DEPUTATION TO MR. CHAPLIN.

A deputation from the Workhouse Infirmary Nursing Association waited upon Mr. Chaplin, President of the Local Government Board, at the House of Commons, on April 15th. The objects of the deputation were as follows:—

1. To ensure the appointment of only trained nurses in workhouse infirmaries.
2. The abolition of pauper help for nursing duties.
3. To define the position of the trained nurse in relation to the untrained matron and to the master of the workhouse, and to consider the means by which this could best be carried out.

Lord Montagu of Beaulieu introduced the deputation, and among those present were: The Lady Wantage, Constance, Marchioness of Lothian, Miss Louisa Twining, Mrs. E. Coysgarne Sim, Mrs. A. C. Powell, Mrs. S. D. Fuller, Miss Wilson (hon. secretary), Mr. H. Bonham-Carter, Dr. Dolan (medical officer of St. Luke's Hospital, Halifax), and Mr. F. Feeney, M.A., J.P. (Guardian of the Poor, Cheltenham).

Lord Montagu of Beaulieu gave a summary of the work of the association during more than sixteen years, in which time over 700 nurses had been appointed, 208 of these having been trained at the expense of the association. He spoke of the difficulties of the nurse's work and position in country workhouses, where the trained nurse is subject to the untrained master and matron, and also dwelt forcibly on the need for the appointment of trained nurses, remarking, "I know instructions are issued by the Local Government Board in favour of trained nurses being employed, but an instruction is one thing, and an order is another." He informed Mr. Chaplin that the deputation desired to ask him to very seriously consider whether he could not make the instructions go further than the Local Government Board has yet done in giving assistance towards the advancement of the objects that the association have in view.

Mr. F. Feeney, M.A., J.P., Guardian of the Poor, Cheltenham, barrister-at-law, took up the second point of the memorandum, stating that all over the country one nurse would be found in charge of sixty, seventy, or even eighty beds—a perfectly impossible task for one woman, however competent and conscientious she might be, to undertake satisfactorily. It was often the case that not only had she to look after these patients, but there was no night nurse at all. Out of forty-seven unions there were twenty-five cases in which there was no trained nurse at all, and thirty-five cases in which there was no night nurse. Taking into consideration that a single-handed nurse has, besides performing her duties, to get out for exercise, so necessary for her in her calling, and occasionally for the observance of religious duties, it would be realized that in many cases the patients must be left absolutely alone. . . . Mr. Feeney dwelt strongly on the evils of pauper assistants, quoting a case which had come under his personal knowledge, in which "out of eight or nine pauper assistants one was nearly blind, all were old with the exception of one, one was ill in another way, and the only one of reasonable age was a woman of weak intellect. These people, who had as much need of care as the unfortunate persons in their charge, were in charge of the sick and dying." Speaking of the idea of economy in the employment of paupers, Mr. Feeney said "The pauper assistant has been proved to be wasteful as to the dietary of his charges; owing to his ignorance he spoils the appliances of the workhouse, and still more is he wasteful because, by his ignorance and lack of attention, he fails to cure many cases which might be cured by skilled nursing." Mr. Feeney alluded to the recent case at Wolverhampton, which has been the subject of severe criticism in the press, remarking that he ventured to say that "there is scarcely any guardian who takes an anxious interest in these matters who could not bring before the President some such case as this." With regard to the difficulty of obtaining superior nurses for workhouse infirmaries, "the real root of the matter," said Mr. Feeney, "is that we cannot get good trained nurses owing to the position, or, rather, lack of position, which they are now asked to occupy in workhouse infirmaries. . . . Trained in her particular work, she is put under a woman who has no knowledge of nursing and who interferes with her in these nursing duties. . . . If we can induce you, sir, in your wisdom, to make alterations in these respects, I can only say that I feel sure you will be conferring an inestimable boon on hundreds of our fellow-creatures—those who have a special call upon our sympathies—the sick, the aged, and the dying in our workhouse infirmaries."

Miss Louisa Twining, dealing with the question of appointing women inspectors, said: "If women are needed as inspectors in factories and other places where women are already employed, it seems to follow that they are as much needed in workhouse infirmaries where a large proportion of inmates are women and children, and where are placed the sick of both sexes. . . . Whether these appointments are to be considered as, or called, sub-inspectors, is immaterial to us, as long as an experiment in this direction is made, and the plan started." Miss Twining dwelt upon the uselessness of circulars, saying that the recent one issued by the Local Government Board had never been alluded to at any of the board meetings at her Union, and it was her opinion that instructions from the central authority should not be permissive, but should take the form of orders. She spoke of the workhouse infirmary of the Union where she is a Guardian, which has 200 patients, and not one trained nurse; and she stated that it was her opinion that it would remain in this condition until further and positive orders were issued by the Local Government Board.

Dr. DOLAN, medical officer of the Halifax Union, spoke on the third point of the memorandum, stating that the means by which the association considered the needed reforms might be brought about would be (*a*) to rescind Article 99, paragraph 5 (page 112, Glen's Consolidated Orders): "That any pauper of the fourth class, whom the master may deem fit to perform any of the duties of a nurse or assistant to the matron, may be so employed in the sick wards, or those of the fourth, fifth, sixth or seventh classes," and also Article 210, number 12 (page 219, Glen's Consolidated Orders), under the "Duties of Matron," "To take proper care of the children and sick paupers, and to provide the proper diet for the same." (*b*) To insure that none but trained nurses shall be appointed in future. (c) That the position of the nurse be assimilated, as far as is practicable, to that of schoolmistress. Dr. Dolan referred to the admirable memorandum drawn up by Dr. Downes and issued by the Local Government Board in 1891, in which the employment of pauper nurses is deprecated, and remarked: "This memorandum is unanswerable, but it is permissive. Some boards of guardians have fallen in with these suggestions, convinced by the facts laid before them, but many have taken little notice of them." He quoted the recent case at West Bromwich Union where the guardians promoted an untrained nurse, a woman of fifty-five years of age, to be head nurse; and when one of the Local Government Board inspectors objected to the appointment, he was answered with the avowal that there was no order of the Local Government Board to appoint a trained nurse, and that the guardians were quite justified in keeping on the woman they had appointed. . . . Dr. Dolan concluded: "Your authority, sir, is an important factor, and we ask you to fully exercise it on this important question. There will never be any advance on this question until an order is issued from the Local Government Board. Public opinion will support you; you will have the unanimous approval of the medical profession and of the medical journals. The Workhouse Infirmary Nursing Association is not asking for the impossible. . . . In the present Poor Law Orders the only qualification insisted upon for a nurse is that she 'may be able to read directions on medicine bottles.' . . . The association has drawn up a striking analogy between the respective positions of the nurse and the schoolmistress under the Poor Law, which I will leave with you for your consideration."

Mr. CHAPLIN, in his reply, dealt with the points set forth by the deputation, and in conclusion remarked: "I can assure you of this, that I am perfectly alive to the desirability of the objects you have in view. I am in full sympathy with them myself, and I am anxious to see them carried out to the best of my ability, and by any means within my power. I think if you carefully examine some of the statements that we have circulated in the instructions issued by the Local Government Board, you will see that we are, at all events, animated by the same views as yourselves." Of Dr. Downes' Memorandum, issued in 1892, in which the evils of pauper nursing are dealt with, Mr. Chaplin said: "We have sent copies of this to every board of guardians in the country. It has been pressed upon their attention, and it is an earnest of the views and desires by which we are animated. . . . You must be perfectly well aware of the opposition there is in many cases to doing anything that is new. There are great difficulties to contend with, but I can assure you of this, being as I am in full and entire sympathy with you upon the questions you have addressed to me this afternoon, I shall do everything in my power that seems to me to be reasonable to give effect to them."

### The First and Best Home Hospital.

WHEN the Home Hospitals' Association was first established at Fitzroy House eighteen years ago it had practically no rivals. To-day it has many nursing home competitors, and competition is keen, but Fitzroy House still holds its own. The applications for admittance last year were more numerous, and the financial success was greater than during any previous twelve months in its history. Still more important is the fact that the number of medical men who send in patients also continues to increase.

From the commencement of its history Fitzroy House has been remarkable for one special characteristic, namely, its home-like characteristics. It was opened, and it continues to exist, solely for the benefit of the patients, to secure to them good nursing, comfortable surroundings, and a peaceful and healthy atmosphere. The quiet cheerfulness of the place is evident to the most unobservant visitor, and is highly valued by the doctors.

Fitzroy House supplied an obvious want; it provided for paying patients advantages hitherto denied them. Nursing homes have sprung up like mushrooms since 1880, and can now be counted by the score in some of the London districts, therefore it speaks well for the Home Hospitals' Association that its position is untouched. It has not advertised itself nor charged exorbitant prices, nor instituted vexatious or harassing innovations, and so it continues to prosper.

Each speaker at the recent annual meeting had something pleasant to say of the kindly care and home influence which pervade the establishment. Complaints are so unknown that this somewhat abnormal condition of things occasionally puzzles the minds of those interested. It is a rule of the association that everything needed for a patient shall be provided for him.

A long acquaintance with the Home Hospitals' Association has put many medical men in a position to judge clearly of the tone of the establishment, and they are unanimous in their approval, and testify to having no anxiety whateve about the patients they advise to enter Fitzroy House. The rooms allotted to their use become their homes for the time, and there is no outward semblance of the hospital to render apartments distasteful to the imaginations of most inexperienced persons. The wards are in fact private apartments, free from intrusion, the home altogether supplanting the hospital, although the two names are officially made use of, and the science of the one is happily blended with the comforts of the other.

"Things are done not only well, but pleasantly at Fitzroy House," said an eminent surgeon, speaking at the annual meeting, "and patients recover all the better for this." The care of children, in itself a test of good nursing, has also been accomplished with special success, and it seems obvious that a high standard of excellence is maintained amongst the nursing staff which reflects the greatest credit on the lady superintendent, Miss Pearson.

### Wants and Workers.

*Help for Gloucester.*—A correspondent is endeavouring to put together a parcel of cast linen to send to the sufferers from small-pox at Gloucester, and writes to ask if any readers of THE HOSPITAL will help by sending contributions in the way of under-linen, especially night-dresses and shirts, for men, women, and children. Address Nurse Winter, 16, Yew Grove, Cricklewood, N.W.

### On Certain Aspects of the Nursing Question as Seen in England and Germany.

By a Certificated Midwife.

### VII.—VIENNA AND PRAGUE.

The following year I carried out my original intention of taking a course of instruction in midwifery at the celebrated school in connection with the University of Vienna. I paid the ordinary fees, and carried away my *Zeugniss* at the end of my three months. I did not sleep in the hospital, though I might have done so had I chosen. But the weather was very hot, and the massive, gloomy old buildings were not attractive to a lover of fresh air. I preferred lodging out; and I remember that in walking to school at six a.m. I always chose the shady side of the street, and dreaded having to cross even a small patch of sunshine.

The *Poliklinik* here was not open to women at that time, the Professor at the head of it having a great dislike to them, as he told me candidly. Dr. Gustav Brann, however, who was *Vorstand* of the training school, was of a different nature, and showed me every kindness, and gave me every opportunity of seeing interesting cases and gaining experience. It was the largest lying-in hospital I had seen. I think the number of beds was over 300, and the pupils were numerous in proportion. But the routine of teaching calls for no comment, as it was similar to what I had already gone through. And of the internal arrangements as to admission of patients, &c., and the domestic economy of the institution I know nothing. The whole building was part of a larger group known as the University Hospital, into which I was not allowed to penetrate, where Professors and students were coming and going all day long.

The next hospital I visited was that at Prague. I did not take a course there, for the simple reason that the pupils are mostly natives, and that therefore the lectures are given in the Czech tongue, which I need scarcely say I do not understand. And though by virtue of the introduction afforded me from Vienna I was allowed to be present during some one or two of the addresses which were given to the few German pupils, and was shown over the whole building, I had to be content with this cursory acquaintance. I mention it here because it struck me as the most beautifully appointed and planned hospital which I had seen anywhere. It was quite new, and must have cost a very large sum. The authorities were proud to explain its arrangements to me, and when I expressed my admiration I remember the naïve reply, "Yes, Bohemia has built it as a pattern for the rest of Europe." We seem to know but little about this nation in England. I fancy, however, that the Bohemians have a great opinion of themselves; and I can only think it a very worthy ambition of theirs to set a good example to the rest of the world, and I wish them every success. I carried away with me an impression of the most perfect attention to details in every department, even down to the contents and arrangement of the midwifery bag, with its medicines and appliances, which every pupil carried away with her when she left the school and began her career. I regretted much my ignorance of the Czech language while at Prague. One regrets much as one looks back on life; but I have never regretted the time and attention I gave to making acquaintance with the Continental system of teaching midwifery. I made passing visits to many smaller schools, but they gave me no fresh light; only one and all confirmed the favourable impression which I first received at Stuttgart of the care taken by each country to train competent pupils.

But I asked myself then, and I ask myself again now that I am recording my impressions for the benefit of others, "How would such training suit English ideas?" In the first place, no Englishwomen are accustomed to such strict discipline as is customary in these training schools; no patient would submit to be regarded as mere material; and certainly no pupil would put up with such autocratic behaviour from her teacher as the women of Germany are accustomed to. When one of their *Herr doctors* opens his mouth to scold he is not particular as to the epithets he uses. I have seen books and other articles shied across a lecture-room at a stupid pupil, and heard language suspiciously like cursing and swearing. "Our doctor is *sehr streng*," they would say one to another, and that was all the notice they took. Some of them trembled if they heard his footsteps in the passage, but they bore no resentment. "Do not you tremble, Fräulein, when you meet the *Herr doctor* on the stairs?" said one of the poor women to me at Stuttgart. "Not I," was my answer, "in England we are not brought up to be afraid." "Ah, but the Fräulein is clever! if she were stupid, like me now, she would shake all over when she was scolded. I was up at four o'clock this morning to learn my lesson, and I hid myself in the wood cellar and prayed to the *liebe Gott* that He would soften the Professor's heart, so that I might not be put to open shame in the class."

Poor Frau Schreivogel! She would rather work in the fields from dawn to dusk during the longest summer day than face the doctor with an imperfect answer, she said. I doubt if her prayers were successful on that occasion, for rebukes and taunts flew about the wards and lecture-room all through the term.

Once I came in for my share, and the occasion was this. He found me sitting reading in one of the wards, and the book was a well-known medical work. He had brought his own copy into the lecture-room that morning in order to show us an illustration of some special point not mentioned in our smaller manuals. As I also possessed a copy I had fetched it to study at my leisure. The sharp eye of our teacher detected it at once, and his wrath rose. "How dare you?" said he, snatching the volume out of my hands. "How dare you go into my room and meddle with my books?" "Herr Doctor," said I, politely, though much astonished, "the book is mine; you make a great mistake in accusing me of meddling." "Then it is no business of yours to read such books," cried he, unabashed; "stick to your manual, a woman cannot understand scientific writings." I accepted the situation, and shut up the volume. But he paid me out for putting him in the wrong. A few days after he asked a difficult question in class which no one could answer. So, after a few taunts, he turned to me (I was always accommodated with a chair at his right hand; I fancied that it was not intended as an honour, but to prevent any of the missiles hitting me, which might have been the case had I sat in the semicircle). "You at least, Fräulein, who read such advanced books, can answer my question." "Herr Doctor," said I, "I am an obedient pupil, and since you wished me to be as ignorant as the rest, I have discarded my studies." This was the only occasion on which we fell out, however.

### The Adelaide Hospital.

From the circumstances which attended and led to the resignation of the late superintendent of nurses at the Adelaide Hospital, South Australia, and the appointment of her successor, and the series of misunderstandings, to say the least of them, which resulted in the institution being left without any proper head of its nursing department, it is clear that difficulties in the management of the institution have been growing for some time. We shall await with interest the arrival of fuller details.

### Everybody's Opinion.

[Correspondence on all subjects is invited, but we cannot in any way be responsible for the opinions expressed by our correspondents. No communications can be entertained if the name and address of the correspondent is not given, or unless one side of the paper only be written on.]

### HOLIDAY HOMES FOR NURSES.

Miss Kate McIntyre writes: Will you oblige me by correcting two inaccuracies which occur in your issue of the 18th inst., concerning the Nurses' Home of Rest at Brighton? "A. E." in her kind remarks concerning the home states that it is at 17, Sussex Square. Our number is 12. Also you state editorially that you believe that admission is limited to members of the Royal British Nurses' Association. This is a mistake. The home is open to all nurses who need rest and fresh air. There is not, and never has been, any connection between this Home of Rest and the Royal British Nurses' Association beyond the fact that both institutions were initiated by our hon. secretary, Mrs. Bedford Fenwick, to whose power of organisation this home owes much of its success.

### THE DIFFICULTIES OF PROVINCIAL MATRONS?

"A Physician to a Provincial Hospital" writes:—Your correspondent's article on "The Difficulties of a Provincial Matron" is surely a strange comment on her new work, on the part, presumably, of a trained nurse. I have yet to learn that "thorns, vipers, and evil winds" abound in provincial hospitals for the benefit of newly-appointed London trained matrons, or that provincial nurses "work like third-rate shopgirls, with an occasional touch of the cheeky barmaid." If she should feel *de trop* in the ward when the "young house-surgeon stalks in" she had better leave it, if she has not the ability to let "her vulgar charges" see that she is mistress of the situation, and not they, if need be. Still more had she better leave the hospital if "in her heart she has a great contempt for doctors, nurses—often treatment." She is approaching her work in the very spirit which will render it impossible. She can only irritate where she should by tact and management conciliate so as to bring about what, in her opinion, is a better condition of things. Your correspondent may have fallen on evil ground in what was apparently her first matronship, and when she had not yet learnt the great lesson—how to rule others, which even the sistership of a "fine London ward" will not and cannot teach; but it is quite certain that the wholesale condemnation she indulges in of the character, nursing, discipline, and staff of provincial hospitals of 120 beds or under is merely a reflection of her own utter incapacity for the post she held or (if it is possibly a case of sour grapes) sought. It has been my lot to come in contact with and to train many nurses in provincial hospitals, and I have invariably found them as good and as noble and high-toned a class of women as those coming out of a large London training school, and to evince quite as much of the *esprit de corps* which your correspondent extols but shows so lamentably a want of. If hers is the true reflection (though I do not believe it and hence regret your article) of the spirit in which London sisters undertake provincial duties, then I for one hope never to see one as matron in my wards. There may be faults in provincial hospitals, I admit, but the tone of your correspondent's article shows there are faults equally grave in the training of London sisters. I enclose my card.—[We published the letter from a "discontented" provincial Matron, although we disagreed with the sentiments it expressed, and quite share the views of "A Physician to a Provincial Hospital." Our policy is to give all sides a hearing when we are assured of the *bona fides* of our correspondents, however much we may dissent from their opinions.—Ed. *T. H.*]

### FEVER NURSING.

"Justice" writes: Will you allow me a space in your "Nursing Mirror," in answer to "Ariadne"? I have had three years' general nursing, and have worked for the Metropolitan Asylums Board for more than two years. The time off duty given is eight hours once a week, and from six until ten once a week, besides four hours for a church pass, and the day once a month. It is true that some of the nurses are taken from—well, not a very refined class, but the greater number are women of a better class, and the nursing will stand on a par with that at any hospital. The eight o'clock pass is used mostly for shopping purposes, and not for promenading the streets. The holidays are better than in most other hospitals, and each nurse has her own bed-room. There will always be found some black sheep everywhere, but that does not condemn the whole nursing staff. The charge nurses are all three years trained and certificated. On careful investigation it will be found that fever hospitals are not so rough as represented to the public.

"Fever Nurse" writes: Having read your article in The Hospital for April 11th on fever nursing, I venture to say "Ariadne's" experience cannot have been of recent date, as things in fever hospitals are not as "Ariadne" would have the public believe. Not only do food and pay compare favourably with other hospitals, but also time off duty. Nurses are allowed twelve hours weekly—which may be taken together or divided up as convenient to the hospital work—and a whole day every month. When on night duty we have from half-past eight to eleven a.m., and two days and two nights in each month. With regard to the class of nurses, "Ariadne" must, indeed, have had an unfortunate experience. The present M.A.B. charge nurses are drawn from some of the best training schools in the kingdom, a three years' certificate of training being necessary before they are eligible to hold an appointment. I find the class of nurses no different to what is met with in general hospitals; the majority belong to the middle-class. Many of the best stay three or four years before going in for general training. Of course, the servant class is represented as in general hospitals. I have had a varied experience in hospitals, both at home and abroad, and am content at present to be one of the despised M.A.B. nurses.

### Appointments.

Deaconesses' Institution and Hospital, The Green, Tottenham.—Miss M. Fleetwood has been appointed to the post of Superintendent of Nursing at this institution. She was trained at the Royal Southern Hospital, Liverpool, afterwards working at the City Hospital, South Grafton Street, Liverpool, as charge nurse, and at the Bradford Infirmary as charge nurse of the children's ward. Miss Fleetwood has also had valuable experience as night superintendent of the Bristol Hospital for Sick Children and Women, and has done private nursing in the South of France. Her testimonials are excellent. We cordially wish her continued success.

House of Recovery and Convalescent Home, Gildersmore, Leeds.—Miss M. E. Parker has been appointed Matron to this home. She was trained at the Royal Infirmary, Newcastle-on-Tyne, and has had considerable experience in fever nursing at the Monsall Fever Hospital, having been promoted there to the post of staff nurse after three years' work. Miss Parker has also held the appointment of head nurse at the City Hospital, Grafton Street, Liverpool, and for the last twelve months has acted as deputy matron at the City Hospital, Park Hill. Miss Parker has excellent testimonials from each hospital at which she has worked, and we congratulate her on well-deserved promotion.

### Where to Go.

Queen's Hall, Langham Place, W.—A "Café Chantant and May Day Revel" is to be held at the Queen's Hall on May 7th, in aid of the Hospital for Incurable Children, 2, Malda Vale. It will be opened at 3.30 p.m. by Princess Edward of Saxe-Weimar. Many well-known people are interesting themselves in this entertainment, for this little Home at Maida Vale takes firm hold of the sympathies of all who know its work. The programme contains many novel features, and bids fair to attract a good audience. There are to be stalls for tea and coffee and flowers, May Day dances by children, &c., "witches" and "Pierrots," and the Ladies' Guitar and Mandoline Band have kindly consented to contribute to the amusements. The doors will close at seven, reopening at a quarter-past eight for a concert and entertainment, of which details are not yet out. Tickets may be had from Miss Nembhard, 40, Devonshire Place, W.; Mrs. Linley Samborne, 18, Stafford Terrace, Kensington, W.; and Messrs. Lacon and Ollier, New Bond Street. For morning or evening performance, 2s.; family tickets, six for 10s.

### For Reading to the Sick.

PEACE.

**Motto.**

Peace hath her victories,
No less renowned than war. —*Milton.*

**Verses.**

Fain would my thoughts fly up to Thee,
Thy peace, sweet Lord, to find;
But when I offer, still the world
Lays clogs upon my mind.
—*Hicke's Devotions.*

Peace is God's direct assurance
To the souls that win release
From this world of hard endurance;
Peace—He tells us—only Peace!
—*Lord Houghton.*

Grant peace on earth, and after we have striven,
Peace in Thy Heaven! —*P. Pusey.*

Still in thy right hand carry gentle peace.
To silence envious tongues. —*Shakespeare.*

They may assault; they may distress;
But cannot quench Thy love to me,
Nor rob me of the Lord my Peace. —*Cowper.*

Would that to me life's changes
Did so with blessings come,
That mercies might, like gales of spring,
Cause some new grace to bloom;
And that the storm which scattereth
Each earth-born hope abroad,
Might anchor those of holier birth
More firmly on my God.

I do not ask, O Lord, that Thou should'st shed
Full radiance here;
Give but a ray of peace that I may tread
Without a fear!
Joy is like restless day; but peace divine
Like quiet night;
Lead me, O Lord, till perfect day shall shine
Through Peace to light. —*A. Proctor.*

**Reading.**

This is the heritage of the children of God; "Peace I leave with you," said our blessed Lord; they have peace in their hearts, though trouble and sorrow are often in the path. With the ungodly it is not so; they may walk in a broad untroubled way, but they have no peace within. Let us, therefore, seek to have peace in our hearts, and leave the way to Him who ordereth all things.—*Heavenly Thoughts.*

There is no true rest of soul and heart in time of trial, until we have come to look beyond second causes and human instruments, and have seen the Hand of our God and Father appointing all in His infinite wisdom and love. Cease as far as possible from vain regrets, and leave all calmly to Him who has promised that all things shall work together for good. To bring our hearts to this may be the very meaning and end of the dispensation.—*Anon.*

To pray your wishes is at once the way of peace. Then, if they are granted, you have a comfort in them as tokens from God; and if they come not, well, "It is the Lord." The next step is not only to pray your wishes, but to pray them in the spirit of submission. "O my Father, if it be possible—nevertheless, not my will, but Thine."—*Christina Rossetti.*

"There is no true peace but in being still in God; in always committing all to Him. And they drink the deepest of those refreshing waters who have so far mastered this truth that He is all to them; that to live, is to serve Him. There is a true finding of our life in thus losing it. . . There is an unspeakable blessedness in knowing that we are in His hands; that He who redeemed us is indeed ours in the covenant of everlasting love."—*Bishop Wilberforce.*

### Notes and Queries.

The contents of the Editor's Letter-box have now reached such unwieldy proportions that it has become necessary to establish a hard and fast rule regarding Answers to Correspondents. In future, all questions requiring replies will continue to be answered in this column without any fee. If an answer is required by letter, a fee of half-a-crown must be enclosed with the note containing the enquiry. We are always pleased to help our numerous correspondents to the fullest extent, and we can trust them to sympathise in the overwhelming amount of writing which makes the new rules a necessity. Every communication must be accompanied by the writer's name and address, otherwise it will receive no attention.

**Queries.**

(20) *Trained Nurses as Stewardesses.*—Can you tell me if trained nurses are carried on the P. and O. Steamship Company's boats, and if so, to whom should application be made for an appointment? I have been asked by two nurses to make inquiries, and know I shall best obtain the desired information through your columns.—*Hospital Secretary.*

(21) *Advice to Wives.*—I should be glad to know of a book of help for wives. I think there must be something more modern and instructive than "Chavasse."—*An Old Nurse.*

(22) *Uniform.*—Can you tell me the name and address of the hospital in or near London where the uniform is as follows: Indoor—turn-down collar and cuffs, cap with streamers; outdoor—the usual cloak and bonnet with long veil? Please send me the "Mirror" containing the answer to my question. I understand the paper is free.—*Clarice.*

(23) *District Nursing.*—Can you tell me the name of a district nursing association which trains nurses without premium? I am told there is one where nurses go for nine months' training.—*Nurse Harriette.*

(24) *Diphtheritic Throats.*—Will you kindly answer the following question? When exudation of false membranes appear on tonsils at first, like small patches, enlarging and coalescing, forming one large patch, spreading behind uvula (not involved), pyrexia temperature the first day, the next three or four usually normal, also pulse; exudation disappearing gradually, seeming to have no after ill effects, more than ordinary tonsilitis, such cases being slightly epidemic. Is it diphtheria? Two qualified doctors have so diagnosed it, two others holding a contrary opinion.—*Anonymous.*

(25) *Buenos Ayres.*—Information wanted with regard to nursing in Buenos Ayres.—*Nurse Dorothy.*

(26) *Books Wanted.*—Will you kindly tell me where I can get the following books, and their price? Copeland's "Dictionary of Practical Medicine," Forbes' "Cyclopœdia of Medicine," and Watson's "Principles and Practice of Physic." I may say I take The Hospital every week, and find in it much that is useful to me. I wish there could be a good training school for male nurses in England. Could not the idea be suggested to some wealthy gentleman who might like to establish such a school?—*Companion.*

**Answers.**

(20) *Trained Nurses as Stewardesses (Hospital Secretary).*—It is becoming increasingly usual for trained nurses to be engaged as stewardesses on the large steamboat companies' vessels. Application to be made direct to the companies, whose addresses will be found in the daily papers. See "The Hospital Nursing Supplement," June 3rd, 1894, p. xcv.

(21) *Advice to Wives (An Old Nurse).*—"The Wife and Mother," by Dr. Westland (C. Griffin & Co., price 5s.), would perhaps be what you want. It is a book in much request now.

(22) *Uniform (Clarice).*—We receive many curiosities in the way of "Queries" in the course of the year, but yours is really more impossible than most. How, in the name of common sense, can a uniform be traced by a description of collars and cuffs, and a "usual cloak and bonnet"? And do you think papers are published in order to be given away wholesale? If you will describe the colour and material of the uniform you are thinking of, both of dress and cloak, we will try to distinguish it. The price of The Hospital is 2d. (postage ½d.).

(23) *District Nursing (Nurse Harriette).*—If you have already received not less than one year's training in a general hospital or infirmary, you had better apply, by letter only, to the secretary, Queen Victoria's Jubilee Institute for Nurses, St. Katharine's Royal Hospital, Regent's Park, N.W., or to the superintendent of the Metropolitan and National Nursing Association, 23, Bloomsbury Square, W.C. If a candidate is approved, six months' training in district work is given by the institute, at the end of which time, if considered suitable, her name is submitted to the Queen as being eligible to be entered upon the roll as a Queen's Nurse. Full particulars of the regulations are given in "How to Become a Nurse." (Scientific Press, 428, Strand, W.C.)

(24) *Diphtheritic Throats (Anonymous).*—It seems very probable that these cases were diphtheritic. The proof would be the occurrence during or after convalescence of symptoms of diphtheritic paralysis. It is to be understood that in no inconsiderable number of cases on the borderland of diphtheria the diagnosis is by no means clear, and can only be made certain by means of a bacteriological examination. This is now made for a fixed fee by the Clinical Research Association, 1, Southwark Street, London Bridge, and by other investigators in the same field.

(25) *Buenos Ayres (Nurse Dorothy).*—You will find articles on "Nursing in Argentina" in The Hospital "Nursing Supplement" for December 16th, 1893, p. civ., and for January 27th, 1894, p. clxvi. English nurses should be warned against going to Buenos Ayres or any other foreign city on the chance of making a living at private nursing, unless they have friends there, or independent means. There is an English nurse at the head of the British Hospital, to whom you might apply for information as to possible work. We shall be glad to help you with suggestions as to outfit if you will write again when your plans are fixed.

(26) *Books Wanted (Companion).*—All the books you name are out of print. When in print they are published by Messrs. Longman. You might obtain them by advertising in the "Publishers' Circular." We are very glad you find The Hospital useful and quite echo your wish with regard to a training school for male nurses. No doubt such an experiment will be tried in time, though at present no one seems inclined to move in the matter.

## Figures and Tables

**Fig 1. f1:**